# Numerical Analysis of Slurry–Crack Coupling in Grouting Repair Process of Multiple Cracks in Concrete Material

**DOI:** 10.3390/ma18112472

**Published:** 2025-05-24

**Authors:** Xiaochen Wang, Wei Li, Mingxiang Shen, Hongtao Wang

**Affiliations:** 1 School of Civil Engineering, Shandong Jianzhu University, Jinan 250101, China; wangxiaochen22@sdjzu.edu.cn (X.W.); s766518935@163.com (M.S.); wanghongtao918@163.com (H.W.); 2 State Key Laboratory of Intelligent Construction and Healthy Operation and Maintenance of Deep Under-Ground Engineering, China University of Mining & Technology, Xuzhou 221116, China

**Keywords:** numerical simulation, grouting material, cracked concrete material, hydromechanical coupling

## Abstract

The interaction between slurry and concrete cracks influences the grouting diffusion process during the grouting repair engineering of cracked concrete material. Compared to a single-cracked concrete material, the slurry–concrete crack interaction mechanism of a multi-cracked concrete material is more complicated. This study employs the Monte Carlo method to generate a crack group with a certain probability distribution for characterizing the random cracks in the concrete material. The cracked concrete material is simplified as a pore–crack medium model, and the continuity equation of the slurry in the model is derived. Furthermore, based on the modified interface layer theory, the coupling effect of the slurry–concrete crack is elucidated, and the variation law of each crack opening in the concrete material under different grouting pressures is examined. When the slurry diffuses in a multi-cracked concrete material, the crack opening changes mainly stem from the crack distribution position and crack tendency. Under the same grouting pressure, four main forms of changes occur in the crack opening: gradual increase along the crack distribution, slow decrease, first increasing and then decreasing, and, basically, unchanged. When the grouting pressure increases, crack groups with the same distribution form have similar crack opening change rules, but the change range varies. Under the same grouting pressure, as the number of cracks in the concrete material increases, the coupling effect of the concrete material becomes increasingly obvious.

## 1. Introduction

As urbanization speeds up, a large number of tunnels have entered the operation and maintenance periods [1]. Due to differences in tunnel design standards and construction techniques, most tunnels exhibit varying degrees of concrete cracks. These cracks often serve as water seepage channels, posing threats to tunnel safety during operation [2,3]. Grouting is the common method for repairing concrete cracks, which can achieve crack filling and repair by injecting cementitious materials into the concrete cracks.

In grouting repair work for concrete cracks, the slurry spreads within the crack channels under injection pressure. During the slurry penetration process, the slurry pressure acts on the crack surface and inevitably induces changes in the crack opening [4], altering the dimensions of the slurry flow channel. These variations in crack channel size, in turn, influence the slurry penetration behavior. This interactive process is termed the slurry–crack coupling behavior during concrete crack grouting. Furthermore, tunnel concrete cracks often develop in groups, with multiple fracture sets mutually intersecting and interconnected, exhibiting a networked characteristic [5]. The slurry penetration behavior in multi-cracked concrete becomes more complex. Understanding the slurry penetration behavior in multi-cracked concrete, particularly the effects of slurry–crack coupling, is crucial for determining grouting repair parameters for multi-cracked concrete materials. Thus, investigating the slurry–crack coupling effect in the grouting repair process of multiple cracks in concrete material is important for guiding grouting engineering practices.

Grouting repairment of concrete cracks is a type of crack grouting within grouting engineering [6]. Scholars have theoretically conducted abundant research on crack grouting. Gustafson et al. [7] established a grouting diffusion model for cracked rock mass under constant grouting pressure with Bingham fluid as the slurry constitutive equation. Zhou et al. [8] examined the reinforcement mechanism of cement slurry via in situ grouting tests to analyze the diffusion mode of the cement slurry, providing effective control parameters for cement slurry grouting. Ruan [9] obtained the slurry flow pattern and constitutive equation under different water–cement ratios through laboratory experiments, and subsequently, they established a grouting diffusion model considering the viscosity of a time-varying slurry. Li et al. [10] established the pressure distribution equation of a cement–water glass slurry in a single plate crack, which was verified through a plate crack grouting simulation test. Taking the rapid solidification slurry as the research object, Zhang et al. [11] established the grouting diffusion model considering the non-uniformity of the spatiotemporal distribution of the slurry viscosity. Based on the diffusion characteristics of the cement slurry, Zhou et al. [12] investigated the influence of various factors on the diffusion radius of the slurry, grouting amount, and concretion strength. Furthermore, Li et al. [13] examined the diffusion mechanism of self-expanding grout in rock cracks. Funehag and Gustafson [14] analyzed the rheological properties of silica sol grouting material and proposed a formula to calculate the diffusion distance of one- and two-dimensional flows of this grouting material. With regard to the coupling theory of cracked rock mass, Fransson et al. [15] proposed a parameter to describe the crack aperture variation. Using this parameter, the hydraulic coupling effect under different grouting methods can be evaluated. Based on the unloading–loading theory of rock mass and the slurry migration equation of plate crack, Zheng et al. [16] established a theoretical model of grouting in a single-cracked rock mass considering the coupling effect of the slurry on a single crack. Using UDEC, Kim et al. [17] presented a numerical model of a slurry flow in a single plate crack, proposed the variation law of the crack aperture along slurry diffusion, and analyzed the gap between the numerical simulation results and the analytical solution. Based on the constitutive model of Bingham fluid, Zhang et al. [18] established a theoretical model of crack grouting, considering the coupling effect of slurry–rock by introducing a two-stage crack deformation control equation, and proposed an algorithm that can fully describe the grouting diffusion process. Yang et al. [19,20] used a numerical method to generate an orthogonal crack network and studied the seepage law of slurry under a crack network. However, most existing studies have focused on the grouting diffusion law of a single crack, including the establishment of the relationship between grouting pressure or grouting rate and slurry diffusion distance, and the discussion of the coupling relationship between the slurry and rock of a single crack. Most studies have not considered the concrete lining with multiple sets of cracks while investigating the coupling effect between the slurry and the concrete during the diffusion of the cracks.

The aim of this study is to investigate the slurry–crack coupling in a grouting repair process of multiple cracks in concrete material, especially the influence of a slurry–crack coupling effect on the concrete crack opening. To clarify this issue, the crack groups that obey a certain probability distribution were first generated based on the Monte Carlo method. Then, the multi-cracked concrete was simplified as a pore–crack medium model, and the continuity equation of slurry in the model was derived. The interface layer theory model was utilized to describe the influence mechanism of the slurry pressure on the crack opening. Finally, the influence of different grouting pressures on the crack opening in the multi-cracked concrete was discussed. The results will provide reference significance for the grouting repairment engineering of concrete cracks.

## 2. Theory and Finite Element Model

### 2.1. Grouting Continuity Equations in Concrete Crack

For any complex hydraulic problem of a multi-cracked concrete, the research object is usually abstracted and generalized into a certain mathematical model. The mathematical model of the multi-cracked concrete hydraulics can be divided into three categories: the equivalent continuum model, the crack network model, and the pore–crack medium model [21]. This study considers a cracked rock mass as equivalent to the pore–crack medium model and makes the following assumptions:

1. The slurry is a homogeneous and incompressible fluid.
2. The diffusion of the slurry in the pore–crack medium is regarded as an osmotic diffusion model, and the influence of the percolation effect during the diffusion process is ignored.
3. The pore medium is homogeneous and isotropic.
4. The slurry pressure is continuous at the junction of the cracked and porous media.
5. Cement slurry can be considered as a Newtonian fluid [22]. The derivation is performed according to the constitutive equation of Newtonian fluids.

(1) τ=μγ

where τ is the slurry shear stress, μ is the slurry viscosity, and γ is the slurry shear rate. γ=−dv/dr.

**Figure 1:**
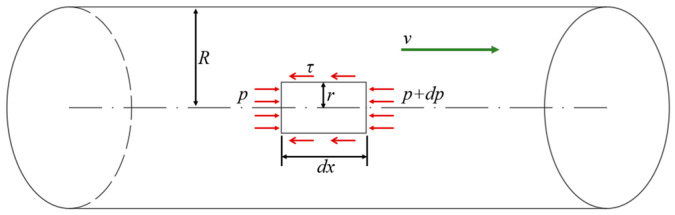
Stress analysis of a slurry in a single seepage channel.

As depicted in Figure 1, for the force analysis of a slurry micro-element in a single seepage channel, the mechanical equilibrium equation can be written as

(2) $$\pi r^{2}dp+2\pi r\tau dx=0$$

Here, r is the micro-element radius, p is the slurry pressure, dp is the slurry pressure increment, dx is the micro-element length, and R is the channel radius.

The shear stress distribution is obtained by simplifying Equation (2):

(3) $$\tau =-\frac{r}{2}\cdot \frac{dp}{dx}$$

Combining Equations (1) and (3):

(4) $$\frac{dv}{dr}=\frac{r}{2\mu }\cdot \frac{dp}{dx}$$

Substituting the boundary conditions and when r=R and v=0, the velocity distribution of the slurry in the seepage channel is as follows:

(5) $$v=-\frac{1}{4\mu }\left(R^{2}-r^{2}\right)\frac{dp}{dx}$$

Therefore, the average flow rate of the slurry through a single seepage channel with radius R is

(6) $$\bar{v}=\frac{1}{\pi R^{2}}\int_{0}^{R}2\pi rv\cdot dr=-\frac{R^{2}}{8\mu }\cdot \frac{dp}{dx}$$

Next, the uniform capillary group model is introduced, and the porous medium is regarded as a capillary array with the same diameter. Using the relationship between the average flow and seepage velocities [23], the seepage velocity of the slurry in the porous medium is obtained as

(7) $$v_{m}=\bar{v}\cdot \phi =-k_{m}\cdot \frac{dp}{dx}$$

where ϕ is the porosity of the injected medium, $v_{m}$ is the seepage velocity of the slurry, and $k_{m}=\frac{\phi \cdot R^{2}}{8\mu }$ is the permeability of the porous medium.

**Figure 2:**
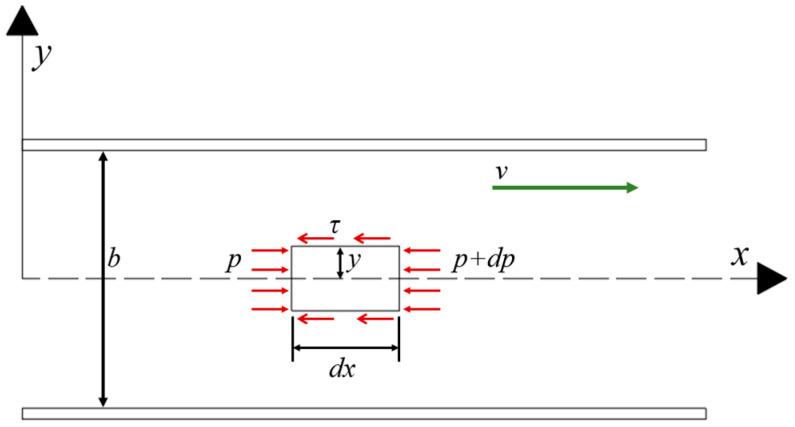
Stress analysis of a slurry in the crack.

Owing to the difference in their structural characteristics, pores and cracks have different permeabilities in the pore–crack medium model. Therefore, a single crack channel is selected to determine the flow characteristics of the slurry in the crack medium. Figure 2 presents the stress analysis of a slurry micro-element in any crack channel.

For any slurry micro-element, the mechanical equilibrium equation is

(8) 2τdx+2ydp=0

Here, y is the slurry micro-unit height and b is the crack opening.

The shear stress distribution of the slurry in the crack channel is obtained by rewriting Equation (8):

(9) $$\tau =-y\cdot \frac{dp}{dx}$$

Combining Equations (1) and (9) yields

(10) $$\frac{dv}{dy}=\frac{y}{\mu }\cdot \frac{dp}{dx}$$

Substituting the boundary conditions and when y=b and v=0, the flow velocity distribution of the slurry within a single crack channel is

(11) $$v=\left(\frac{b^{2}-4y^{2}}{8\mu }\right)\cdot \left(-\frac{dp}{dx}\right)$$

Then, the average flow rate through the crack channel is as follows:

(12) $$\overset{-}{v}=\frac{1}{b}\int_{-\frac{b}{2}}^{\frac{b}{2}}v\cdot dy=\frac{b^{2}}{12\mu }\cdot \left(-\frac{dp}{dx}\right)$$

Equation (12) can be rewritten as

(13) $$v_{f}=\overset{-}{v}=-k_{f}\cdot \frac{dp}{dx}$$

where $k_{f}=\frac{b^{2}}{12\mu }$.

The continuity equation for the slurry flow in the pore crack is as follows:

(14) $$\frac{\partial }{\partial t}\left(\rho \cdot \phi \right)+\nabla \left(\rho \cdot v\right)-q\cdot \rho =0$$

where ρ is the slurry density, ϕ is the porosity of the medium, and q is the strength of the source.

The continuity equation of the slurry flow within the pore–crack medium is obtained by combining Equations (8), (13) and (14):

(15) $$\phi_{f}\cdot \frac{\partial p_{f}}{\partial t}-\frac{k_{f}}{\mu }\cdot \nabla^{2}p_{f}-q=0$$

(16) $$\phi_{m}\cdot \frac{\partial p_{m}}{\partial t}-\frac{k_{m}}{\mu }\cdot \nabla^{2}p_{m}-q=0$$

Here, $\phi_{f}$ and $\phi_{m}$ are the porosities of the crack and pore media, respectively. Additionally, $p_{f}$ is the slurry pressure within the crack medium, and $p_{m}$ is the fluid pressure within the void medium.

### 2.2. Description of Crack Opening Based on the Interfacial Layer Model

The concrete crack is an interface with certain characteristics formed under certain causes, including stress and seepage corrosion. Concrete cracks usually have a certain extension scale and thickness. In terms of structural characteristics, they are obviously different from a complete concrete block. The concrete crack is not a pure void; it mainly consists of two surfaces and the solid, liquid, and gas phases present between them, and it has complex material composition and structural characteristics. To facilitate the analysis of the mechanical properties of concrete cracks and the seepage coupling mechanism in cracks, based on the above characteristics, the concrete crack can be abstracted as a layer in the middle of a complete concrete block, which is called the interface layer [24]. For a general unfilled hard structural plane, the interface layer is a layer enveloped by the two walls of the structural plane, while for a filled structural plane, the interface layer includes the two walls as well as the fillings and structural belts present on the two walls.

According to a previous study [24], the crack deformation analysis using the interface layer model is usually treated as a plane strain problem. Under hydraulic coupling, the normal deformation u of the interface layer can be divided into two parts: displacement $u_{1}$ under the action of normal stress and displacement $u_{2}$ under the action of dilatancy or shear contraction, i.e.,

(17) $$u=u_{1}+u_{2}$$

The stress state and normal deformation of the interface layer can be expressed as follows:

(18) $$b=b_{0}-u=\left(1-\chi \right)b_{0}$$

where b represents the crack opening, $b_{0}$ represents the initial crack opening, and χ is a constant with a dimension of 1, which can be expressed as follows:

(19) $$\chi =\frac{A_{0}+B-2AB}{1-A_{0}B}$$

Here,

(20) $$A_{0}=1-e^{-\frac{\sigma_{n}}{\lambda +2G}}$$

(21) $$B=1-e^{\frac{1}{2G}\left[\left(\arctan\frac{\left|\tau \right|}{s}-\varphi \right)\left|\tau \right|-\frac{s}{2}\ln\left(1+\frac{\tau^{2}}{s}\right)\right]}$$

where $\sigma_{n}$ is the normal stress, τ is the shear stress, λ and G are the Lame constants, and s and φ are the shear parameters.

Considering the actual grouting project, the coupling effect of the slurry–concrete crack mainly stems from the change in the normal stress caused by the slurry pressure [25]. The shear stress change in the interface layer and the shear deformation can be ignored. Combining Equations (18)–(21), the simplified crack opening can be expressed as

(22) $$b=b_{0}\cdot e^{-\frac{\sigma_{n}}{\lambda +2G}}$$

For the pore–crack medium model, the crack force should comprise the fluid and concrete crack forces on both sides of the crack. Therefore, when Equation (22) is used for calculation, the normal stress $\sigma_{n}$ can be approximately expressed by force $\sigma_{0}$ on both sides of the concrete crack and slurry pressure p. Consequently, Equation (22) is rewritten as

(23) $$b=b_{0}\cdot e^{-\frac{\sigma_{0}-p}{\lambda +2G}}$$

Equation (23) shows that the crack aperture is a function of the difference between the fluid and concrete forces on both sides of the crack. Force $\sigma_{0}$ of the concrete crack on both sides of the crack can be considered constant, and as the fluid diffusion progresses, fluid pressure p acting on the concrete crack continuously changes, and the crack opening accordingly varies.

### 2.3. Finite Element Model of Multi-Cracked Concrete Grouting

The Monte Carlo method is a random simulation method that provides approximate solutions [26]. Based on the Monte Carlo method, the prior probability model of crack characteristic parameters is determined. Then, the model is randomly sampled to determine the specific parameters of each crack. The trace length and tendency of the cracks are essential parameters for simulating two-dimensional random cracks [21]. According to the literature [21,26,27,28,29], the crack trace length and crack dip angle can be considered to obey the negative exponential and normal distributions, respectively. Table 1 presents the specific parameter values.

**Figure 3:**
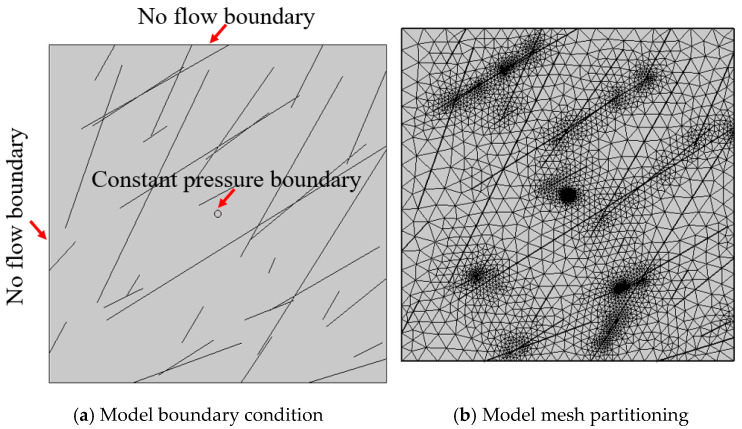
A grouting model of multiple cracks.

Based on the parameter values in Table 1, in the 3 m × 3 m research area, a crack group obeying a certain distribution is generated through MATLAB R2020b programming, which is imported into the finite element software COMSOL multiphysics 5.5 for calculation. Considering the boundary effect, the area of 1 m × 1 m is finally employed for calculation and analysis. Figure 3 shows the model boundary conditions and mesh division of the grouting model. The grouting hole is assigned a constant pressure boundary condition, while the model boundaries are defined as no-flow boundary conditions. The computational region is meshed using triangular elements, with local mesh refinement applied at each fracture location. The maximum and minimum mesh sizes were 6.7 × 10^−2^ m and 3.4 × 10^−4^ m, respectively. The calculation was conducted using the MUMPS solver built into the COMSOL software. A mesh-independence study ensured computational accuracy. Additionally, solver convergence was systematically verified by monitoring equation residuals. Table 2 lists the calculated parameters of the model.

The governing equation of the entire model calculation domain is the pore–crack medium model introduced earlier. The deformation characteristics of each crack in the study area are expressed according to Equation (23) in the interface layer theory described earlier.

## 3. Results and Discussion

### 3.1. Calculation Results and Analysis Under Certain Crack Density

**Figure 4:**
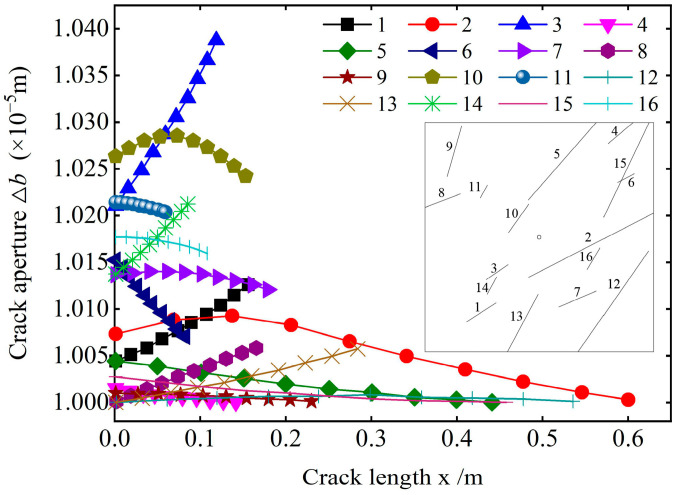
Variations of the crack aperture along its length.

Figure 4 depicts the variation of the crack opening along the crack length. The crack opening significantly varies from the beginning of grouting to the spreading of the slurry to the boundary. The variation degree of the crack opening at different distribution positions is different, and the opening of all the cracks is nonlinear along the length direction. Based on the variation degree of the crack opening and the change form along the length direction, the crack opening variation in the study area can be divided into four types, as listed in Table 3 (Types of aperture variations).

The distribution position of cracks in the study area shows that the distribution position of cracks and the dip angle of cracks are the main reasons for the four types of opening changes. The slurry is injected from the grouting hole and radially diffuses to the boundary. Under the influence of the formation resistance and slurry viscosity, the diffusion velocity and penetration pressure of the slurry gradually decay with increasing diffusion distance, resulting in the spatial inhomogeneity of the slurry pressure during the diffusion process. The randomness of the crack distribution leads to the significant variation of the pressure on the crack at different distribution locations and any crack along its length, consequently resulting in the above distribution of the crack in the study area.

Combined with the analysis of Figure 4, the dip angle distribution of cracks in type A aperture variation is similar, and one end of the crack points to the grouting port, which causes the pressure distribution on the crack to have certain similarities when the slurry migrates in the crack. The slurry pressure acting on the crack is higher, and the crack opening variation is obviously closer to the grouting hole. Therefore, from one end of the grouting hole to the other end, the crack opening gradually increases until it reaches the maximum value. The crack aperture variation trend in type B is opposite to that in type A. Additionally, the crack aperture variation in type C along the length direction is obviously different from that in other types. Due to the slurry diffusion mode and the crack distribution direction, the slurry does not simultaneously diffuse to the entire crack. However, a sequence of events occurs. Under the influence of factors such as the injected medium and the properties of the slurry itself, the slurry pressure decays along the crack during the diffusion process. Thus, the slurry pressure appears at a certain position when it acts on the crack, and the pressure gradually decreases at both ends, with the maximum value at the center. Consequently, the crack opening first decreases and then increases. In contrast, type D aperture variation cracks are mostly located near the boundary of the study area and are far away from the grouting hole. When the slurry diffuses to this position, the pressure attenuation is large, resulting in a small pressure on the slurry along the crack, which causes the crack extension opening to change slightly. Therefore, the crack opening of this type does not vary significantly. Hence, in actual projects, the reinforcement and water gushing control effects are relatively poor for cracks located far from the grouting hole.

### 3.2. Sensitivity Analysis of Crack Aperture Under Different Grouting Pressures

To determine the influence of different grouting pressures on the crack opening in the same crack density area, the crack in type A is taken as the research object, and the crack opening changes in the study area under three grouting pressures are calculated.

**Figure 5:**
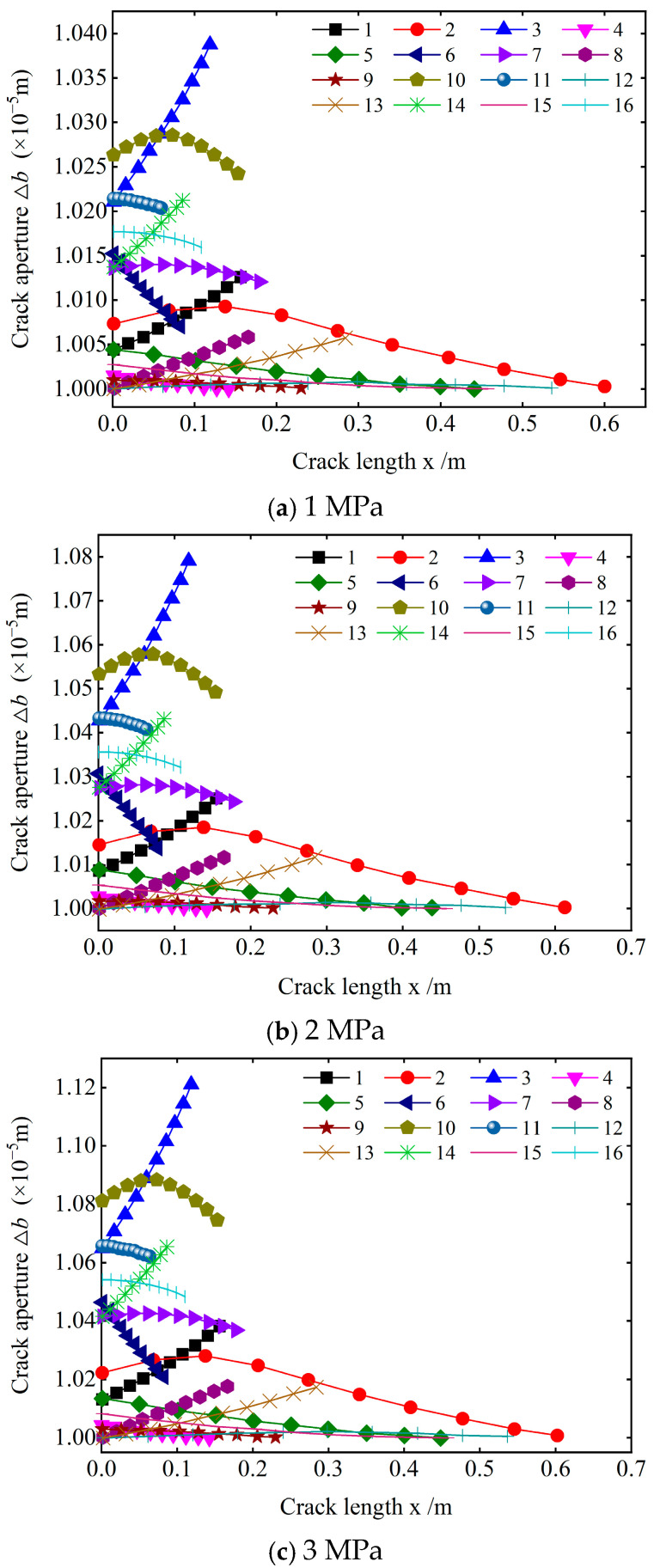
Aperture variations under different grouting pressures.

Figure 5 exhibits that the crack opening generally increases with the grouting pressure for cracks with the same initial openings and distribution positions under different grouting pressure conditions, although differences exist in the opening variation of a crack along its length direction.

As stated earlier, the variation of the crack opening in the study area can be divided into four categories. Taking cracks 1, 16, 10, and 4 as examples of these four categories, the influence of different grouting pressures on the variation of the crack opening of the four types of cracks is analyzed.

**Figure 6:**
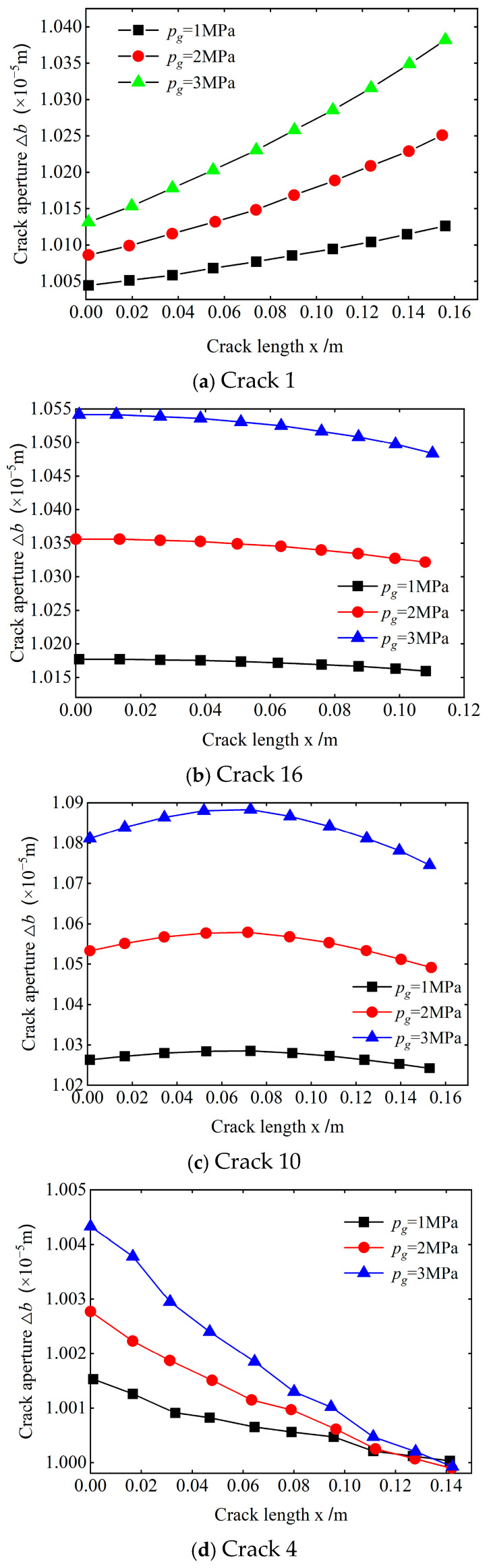
Aperture distribution of typical cracks.

Figure 6 illustrates that as the grouting pressure increases, the variation law of the crack opening along the length direction remains basically unchanged, but the increment of the opening per unit length of the crack obviously changes. For the four types of cracks, the opening increment per unit length of the crack under high grouting pressure is significantly greater than that under low grouting pressure. Comparison denotes that when the extension direction of the crack is approximately parallel to the slurry flow direction, the change in the crack opening per unit length under high grouting pressure is significantly greater than that under low grouting pressure near the grouting port. On the other hand, near the boundary position, the change in the crack opening per unit length under high grouting pressure is not significantly different from that under low grouting pressure. When the crack distribution direction is approximately perpendicular to the slurry flow direction, the change in the crack opening per unit length under different grouting pressures is not significant.

### 3.3. Calculation Results and Analysis Under Different Crack Densities

**Figure 7:**
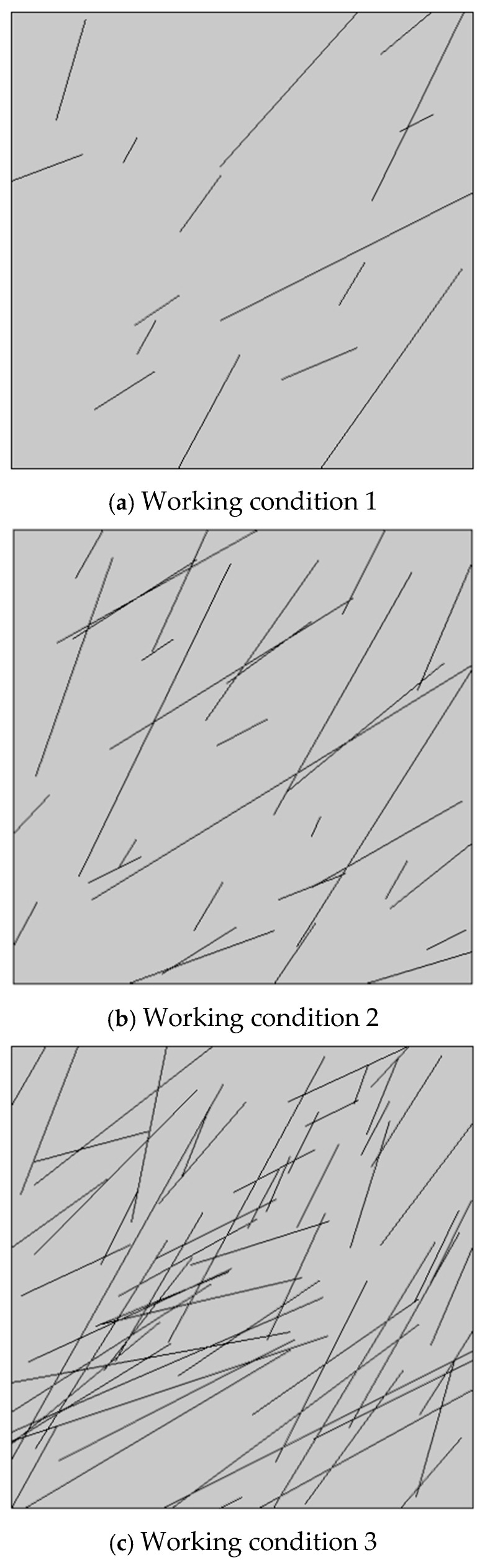
The distribution of cracks in the three cases.

Computational analysis is conducted to determine the crack opening variation under the same grouting pressure conditions for cracked concrete with different crack numbers. Due to the large number of cracks in the area under working conditions 2 and 3, the average crack opening increment of the entire crack is used as the evaluation index for calculating the average crack opening increment of each crack under three different crack densities to elucidate the influence of different crack densities (shown in Figure 7). The average crack aperture increment can be calculated as follows:

(24) $$\Delta \bar{d}=\frac{1}{m}\int_{0}^{m}\left(d_{0}-d\left(x\right)\right)dx$$

Here, $\Delta \bar{d}$ is the average opening increment of crack, $d_{0}$ is the initial opening of the crack, $d\left(x\right)$ is the distribution function of the crack opening along the length direction, and m is the crack length.

**Figure 8:**
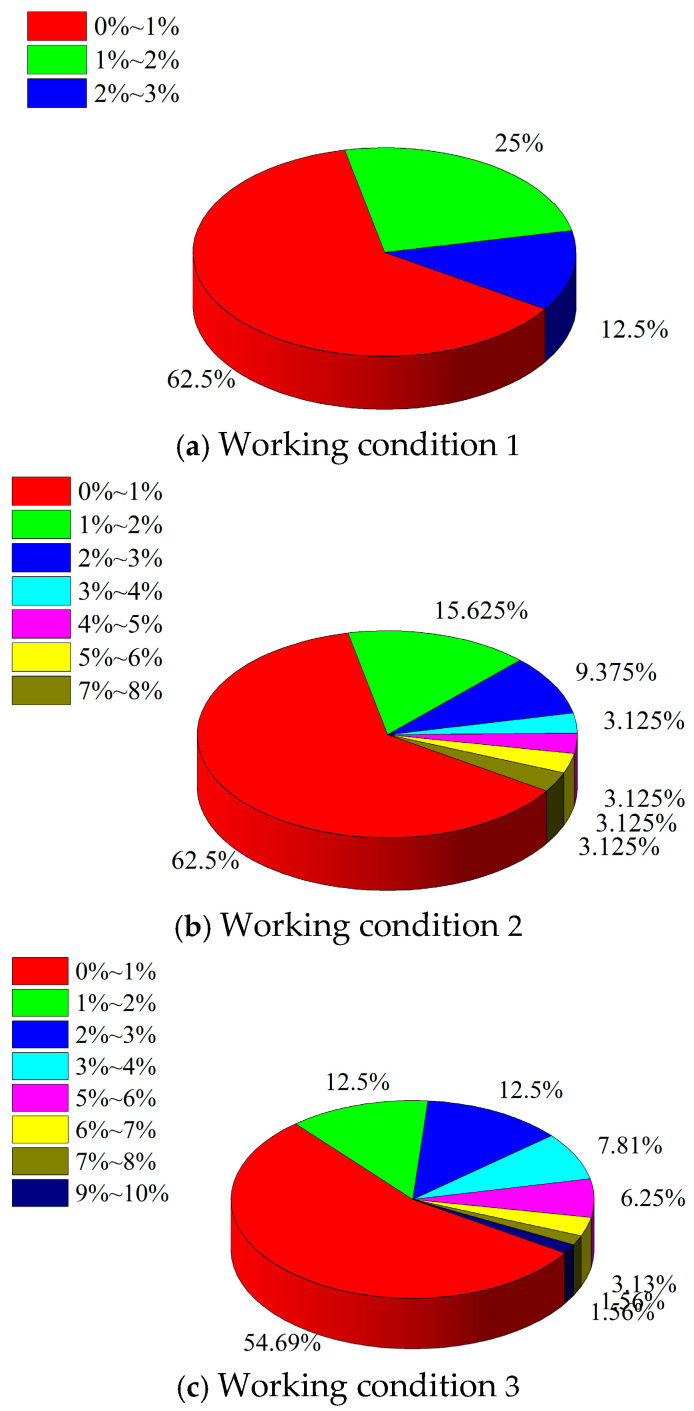
Percentages of the aperture increment of each crack in the three cases.

By comparing the three working conditions, Figure 8 shows that the distribution range of the increase in the crack opening in the region is related to the number of cracks in the region. Furthermore, the distribution range of the increase in the crack opening increases with the number of cracks in the region. This indicates that the number of cracks in the region increases, and the cracks in the region are more likely to lead to a large deformation. Under the three working conditions, the maximum increment of the crack opening does not exceed 3%, 8%, and 10% of the original crack opening. Moreover, the average opening increment of more than 50% of the cracks is within 1%. This signifies that the number of cracks in the region is an important factor influencing the crack opening variation. As the number of cracks in the region increases, the cracks become more prone to deformation. Simultaneously, the crack opening variation increases with the number of cracks in the region. As the number of cracks in the cracked concrete increases, the strength parameters of the cracked concrete gradually decrease. Therefore, under the same grouting pressure, the influence of the coupling of the slurry–concrete crack becomes more obvious.

### 3.4. Discussion

Grouting is a primary approach for repairing concrete cracks. Under the grouting pressure, the slurry spreads in the cracks. During this process, the slurry pressure inevitably causes the crack opening to increase. The variation of crack opening alters the flow channel dimensions of the slurry, which, in turn, affects its spread. It is a hydromechanical coupling problem. However, constrained by the limitations of existing experimental technology, current test devices for concrete crack grouting repair struggle to simultaneously monitor crack deformation and the associated changes in grout pressure and flow velocity field caused by opening variations. This has made the hydromechanical coupling issue in concrete crack grouting repair a research challenge in experimental simulation. In future research, we will focus on addressing this issue to further improve this study.

On the other hand, there is the filtration phenomenon in the slurry penetration process in the pore–crack medium. The fine particles in the slurry can permeate through the pores, while large particles cannot permeate through the pore structure. For concrete, the size of its inherent pore structure depends on the degree of internal erosion. Concrete with a low degree of internal erosion has smaller pores and is less likely to experience filtration. In contrast, concrete with a high degree of internal erosion has a larger pore structure and a more pronounced filtration effect. This study mainly focuses on the situation where the internal pore structure of concrete is relatively small. Therefore, the filtration effect is ignored in the model assumptions. The conclusions drawn in this paper are also based on this condition. Of course, if the filtration effect is taken into account, the research results may differ. This is also a direction for future research.

## 4. Conclusions

(1) When slurry is injected from a grouting hole to a certain position, four kinds of variation rules exist regarding the direction of the crack opening extension: gradual increase along the crack length; gradual decrease; first increasing and then decreasing; and, basically, remaining unchanged. This variation mainly depends on the distribution location and production status of the cracks.
(2) As the grouting pressure increases, the variation law of the crack opening extension remains basically unchanged, but the crack opening per unit length obviously varies. Specifically, the increase in the crack opening per unit length under high grouting pressure is obviously greater than that under low grouting pressure. Along the crack distribution direction, the variation of the crack opening per unit length caused by different grouting pressures mainly exhibits four trends: gradual increase along the distribution direction; gradual decrease; first increasing and then decreasing; and, basically, remaining unchanged.
(3) The average opening increment distribution of cracks obviously varies with the number of cracks in the region. The range of crack opening increment increases with the number of cracks in the region, but it does not exceed 10%. The number of cracks in a cracked concrete determines the size of the cracked concrete affected by the slurry–concrete crack coupling.

## Figures and Tables

**Table 1:** Calculation case.

Type 1	Dip Angle/(°)		Trace Length/(m)	Density of Crack (Strips/m^2^)
	Mean Value	Variance	Mean Value	
1	28°	6.6	0.37	16
	62°	4.3	0.34	
2	28°	6.6	0.37	32
	62°	4.3	0.34	
3	28°	6.6	0.37	64
	62°	4.3	0.34	

**Table 2:** Parameters for modeling.

Parameters	The Value
Porosity of porous media	0.22
Permeability of porous media	4 × 10^−8^ m^2^
Elastic modulus	5 × 10^2^ MPa
Poisson’s ratio of concrete	0.2
Crack porosity	1
Slurry density	2940 g/cm^3^
Slurry viscosity	0.08 Pa·s

**Table 3:** Types of aperture variations.

Type	Characteristics of a Change in Opening	Typical Cracks
A	① The crack opening gradually increases along the distribution direction. ② The crack opening greatly varies along the distribution direction.	1, 3, 8, 13, 14
B	① The crack opening gradually decreases along the distribution direction. ② The variation of the crack opening is small. ③ The variation degree of the crack opening is obviously different, but the overall trend is the same.	5, 6, 11, 15, 16
C	① The crack opening first increases and then decreases along the distribution direction. ② The variation degree of the crack opening is large.	2, 7, 10
D	① There is no obvious change in the crack opening along the distribution direction. ② The variation degree of the crack opening is small.	4, 9, 12

## Data Availability

The raw data supporting the conclusions of this article will be made available by the authors on request.
